# Supplementary material for: Light- and bias-induced structural variations in metal halide perovskites
Source: Nat Commun. 2019 Jan 25;10:444. doi: 10.1038/s41467-019-08364-1 (PMC6347646; doi:10.1038/s41467-019-08364-1)
Supplement: Supplementary file 1 — Supplementary Information [file 41467_2019_8364_MOESM1_ESM.pdf]

# Supplementary Information

# Light and Bias Induced Structural Deformation in Metal Halide Perovskites

Kim *et. al.*

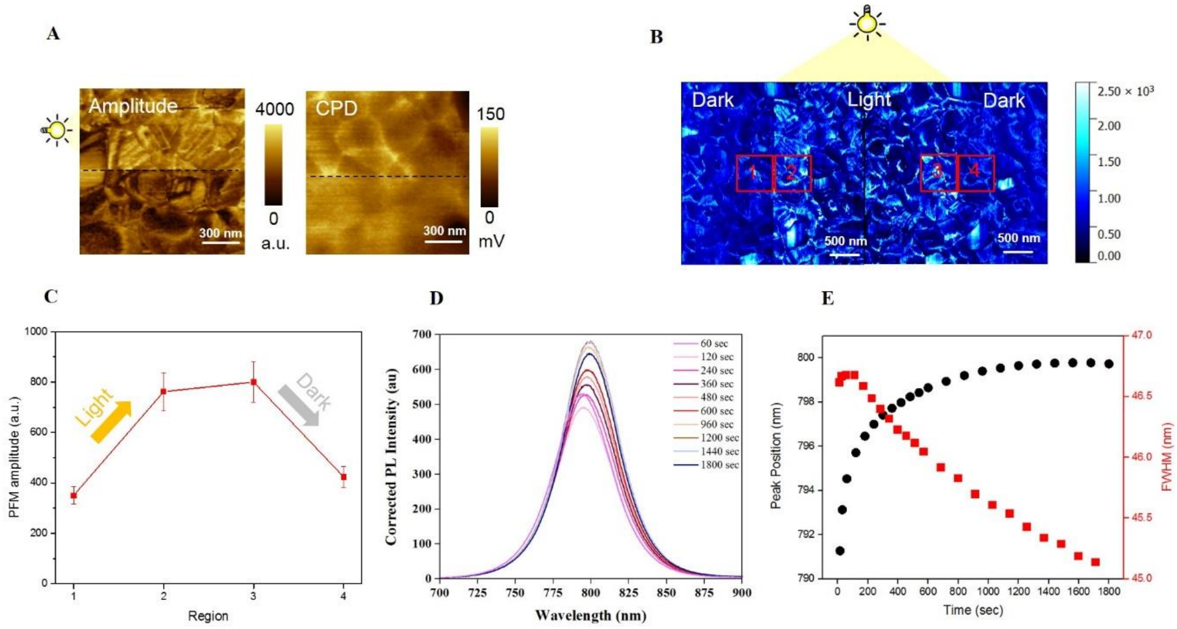


**Supplementary Figure 1. PFM, CPD, and PL measurement over time** (A) Comparison of PFM magnitude and CPD maps. Light is turned on half way through the measurement. (B) PFM amplitude maps under dark and light illumination and (C) a plot of average amplitude at selected areas. (D) Time-evolution of photoluminescence under constant illumination. PFM measurement is carried out over time. (E) Variation of peak positions and FWHM in photoluminescence spectral measurement as a function of time. PL measurement is performed using a 630 nm LED as the light source and a CCD camera as the detection mean. Parasitic emission from inactive layers was removed from the total device luminescence emission. Signal emitted from the device was collected through a band pass filter, 750-850 nm.

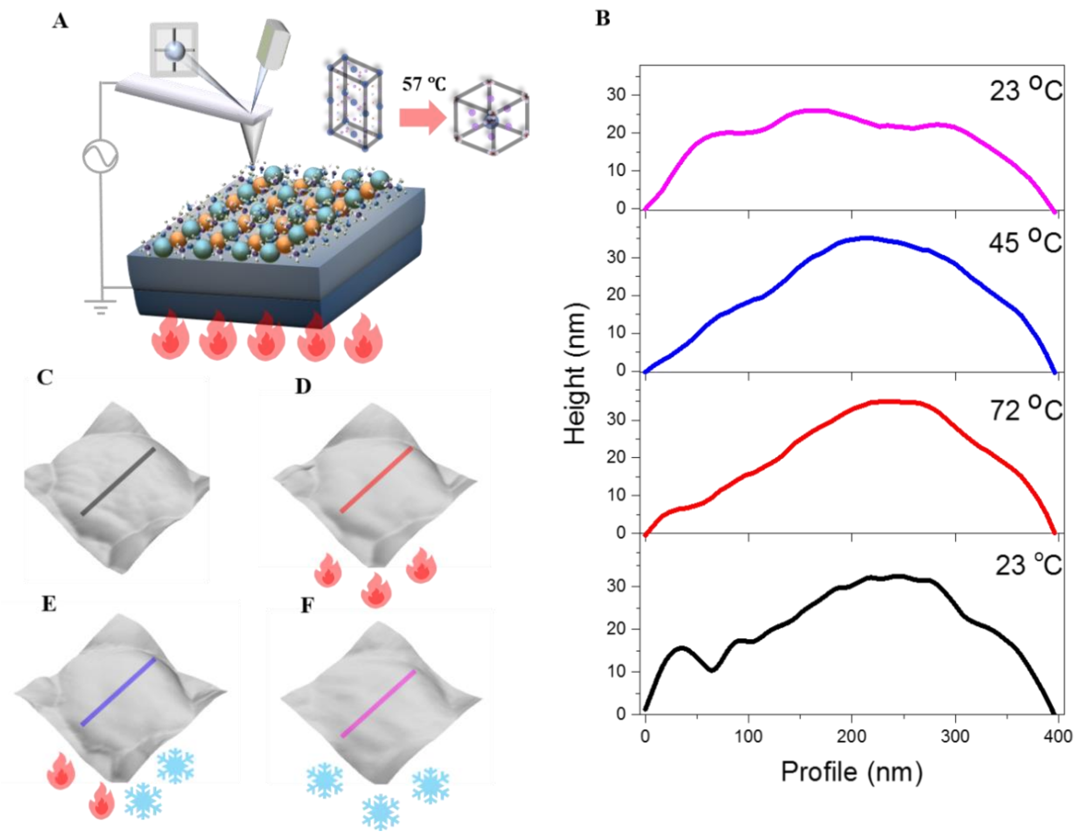


**Supplementary Figure 2. Structural variation on film surface with temperature.** (A) Schematic illustration of AFM work with heater and the film surface transformed from tetragonal to cubic (B) Line profile of the black, red, blue and purple lines in (C to F) Apparently it can be seen that corrugated surface at room temperature could be transformed to smooth surface from 45 ℃, and ,from subsequent measurement, such uneven surface reappears when back to room temperature again (C) 3D Topography in non-contact mode in dark at 23℃ under air, (D) at 45 ℃ (E) at 72 ℃ (F) and back to 23 ℃.

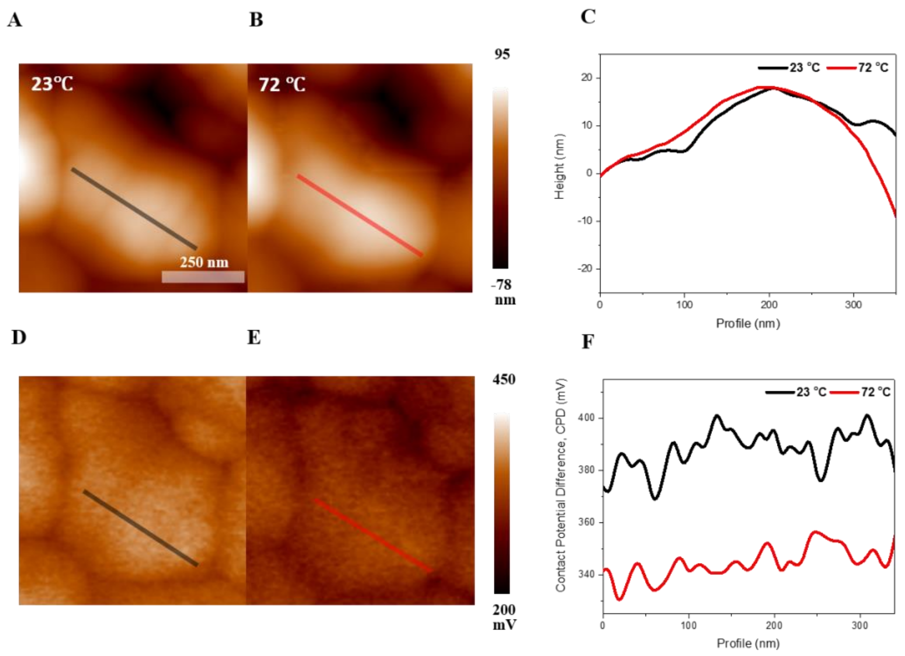


**Supplementary Figure 3. Temperature dependent KPFM measurement.** (A) Topography at 23℃ (B) at 72℃ (c) and line profile of black and red lines in (A to B) the striation patterns disappear with heating up to 72℃ as the film surface undergoes phase transition from tetragonal to cubic. (D) Contact potential difference image at 23℃ (E) at 72 ℃ at the same regions. (F) The line profile of black and red lines in (D to E) striation patterns almost were diminished over 72 ℃ and line profile also shows CPD values were constant at 72℃.

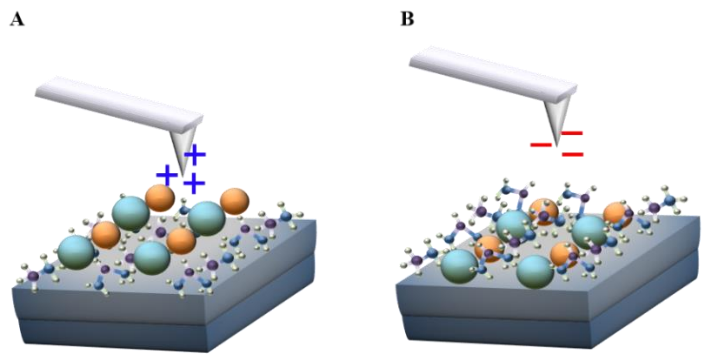


**Supplementary Figure 4. Ionic movement behaviour with charged AFM tip.** (A) shows halide ions' movement to the top surface when applied positive biases and (B) organic ions' movement to the top surface when applied negative biases.

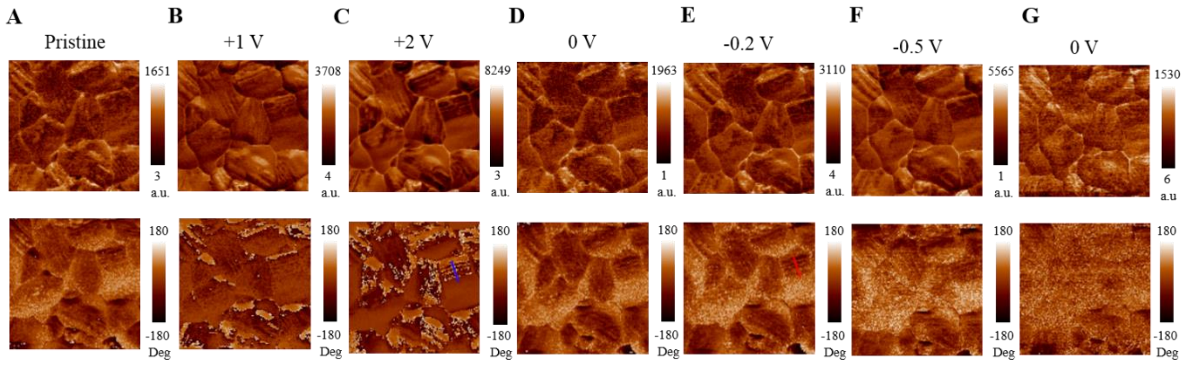


**Supplementary Figure 5. Bias-dependent PFM images of MA-FA mixed halide perovskite in dark condition.** (A to G) shows bias-dependent out-of-plane amplitude and phase maps of metal halide perovskite in dark condition. (A) out-of-plane amplitude and phase images in pristine condition (B to C) when applied positive bias, + 1 and + 2V (D) after back to zero (E to F) when applied negative bias, -0.2 and -0.5 V (G) after back to zero again. Every PFM image was taken on surface of the sample in dark condition. All measurements were performed under ambient conditions using PtIr coated tips. Stripe domains were gained with PFM in contact of AFM tips. Out-of-plane amplitude and phase maps indicate the existence of stripe domain structures.

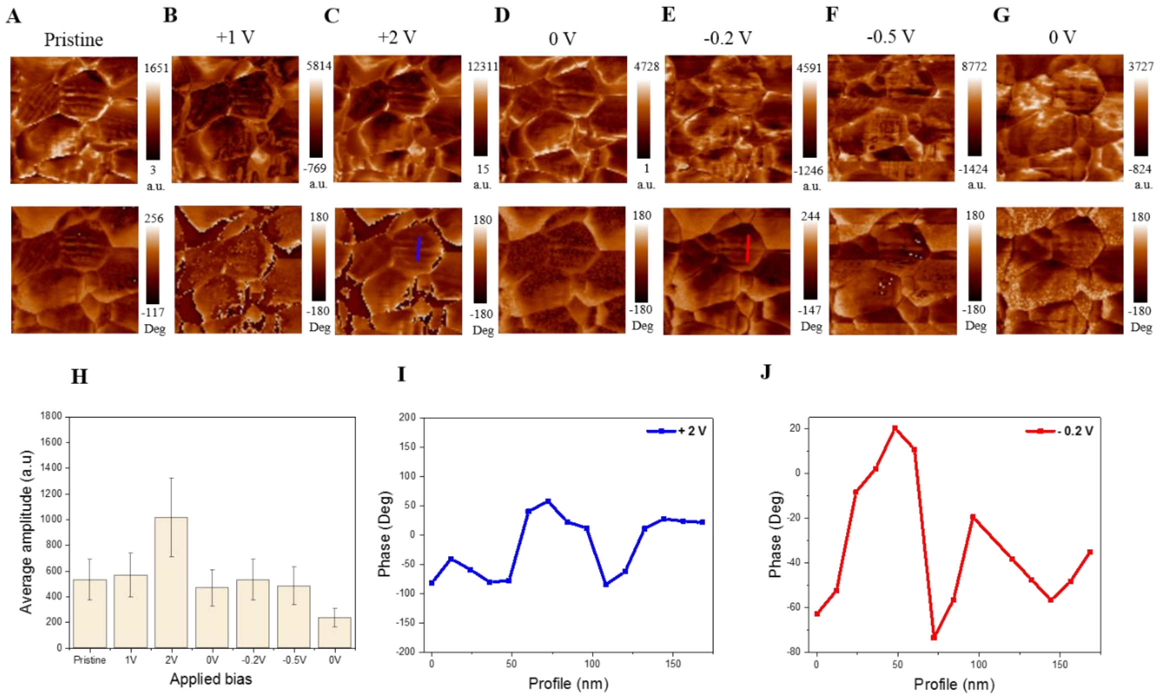


**Supplementary Figure 6. Bias-dependent PFM images of metal halide perovskite in light condition.** (A to G) shows bias-dependent out-of-plane amplitude and phase maps of metal halide perovskite in light condition. (A) out-of-plane amplitude and phase images in pristine condition (B to C) when applied positive bias, + 1 and + 2V (D) after back to zero (E to F) when applied negative bias, -0.2 and -0.5 V (G) after back to zero again. Under illumination, PFM signals were basically higher than in the dark condition. Appearance of stripe domains with light become much strong than no light condition. Likewise Figure S5, out-of-plane amplitude and phase maps show clear striation patterns as a function of applied biases, especially as for positive bias. (H) Graph of average amplitude signals with biases. (I) The line profile of blue line in Figure S6C (J) The line profile of red line in Figure S6E. Overall tendency with biases is the same with dark condition. It is obvious that switched patterns in phase signals were shown in domains although their values are relatively lower than dark condition.

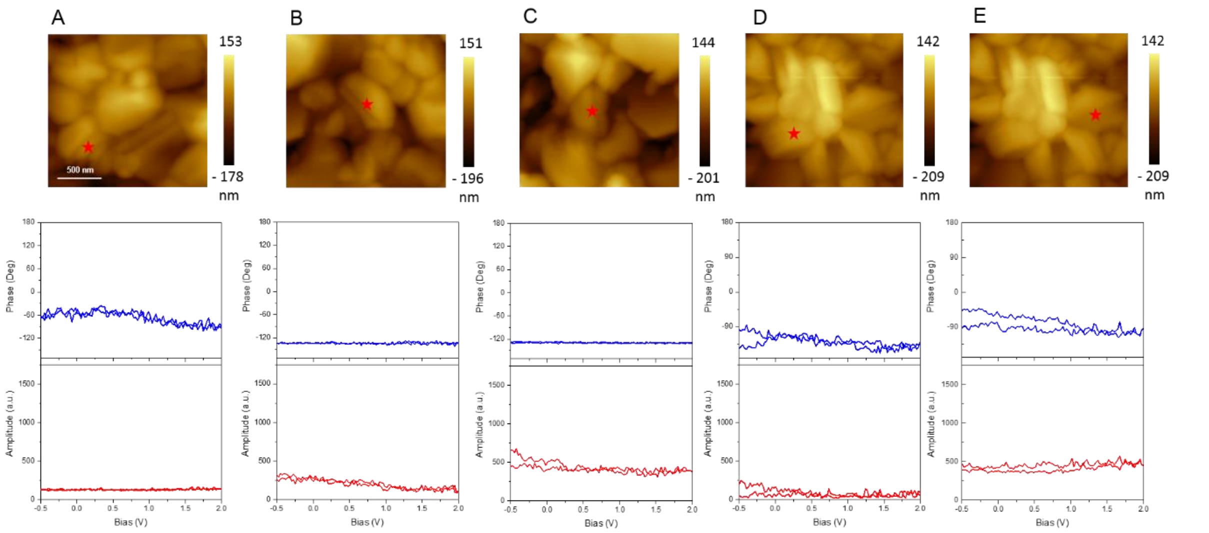


**Supplementary Figure 7. Polarization-electric field hysteresis loop measurement at an inner grain.** (A-E) Topography image, and phase and amplitude signals as a function of biases measured in the location marked by the red star in topographic images. To check ferroelctricity of metal halide perovskites, the out-of-plane phase and amplitude signals as a polarization-electric field hysteresis loop measured with applied biases from -0.5 to 2V of 5 V/s. There exists no butterfly curve in both amplitude and phase. In other words, the steady values in phase suggest no ferroelectricity in mixed halide perovskite films.

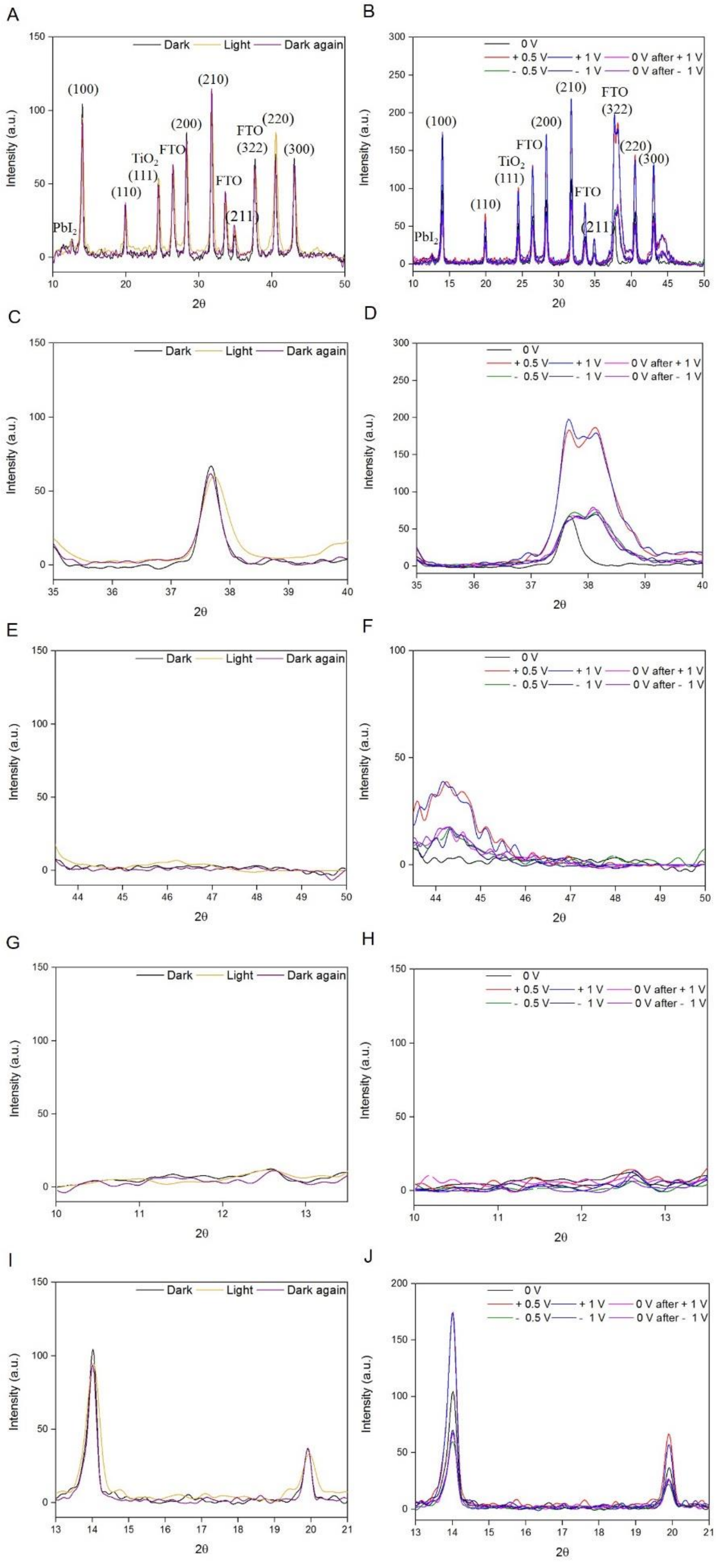
A
Dark
Light
Dark again
(100)
(110)
$TiO_2$
(111)
FTO
(200)
(210)
FTO
(322)
(220)
(300)
FTO
(211)
$PbI_2$
Intensity (a.u.)
2θ
B
0 V
+ 0.5 V
+ 1 V
0 V after + 1 V
- 0.5 V
- 1 V
0 V after - 1 V
C
D
E
F
G
H
I
J

**Supplementary Figure 8. Overlaid XRD patterns.** (A) Overlaid XRD patterns under dark and light illumination, (B) and with positive bias up to + 1 V, and then back to zero bias, and with negative bias to – 1 V, and then back to zero bias. (C) Zoom-in of the peaks near 38 degrees with light (D) and bias effect. (E) Zoom-in of the new peak near 45 degrees with light (F) and bias effect. (G) Zoom-on the $PbI_2$ peak near 13 degrees with light (H) and bias effect. (I) Zoom-on the (100) and (110) peaks near 14 degrees and 19 degrees with light (I) and bias effect (J)

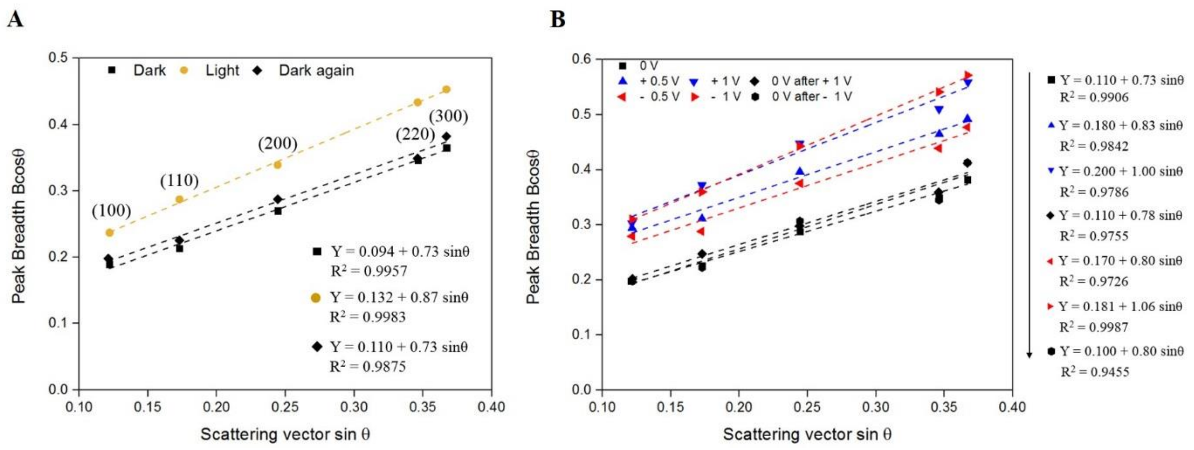


**Supplementary Figure 9. Photo-induced and bias-dependent Williamson - Hall plot.** (A) Williamson – Hall plot from the XRD peak full width at half maximum (FWHM) under dark and light illumination and then dark again (B) with positive bias up to +1 V, and then back to zero continuously with negative bias to – 1 V, and back again to zero.

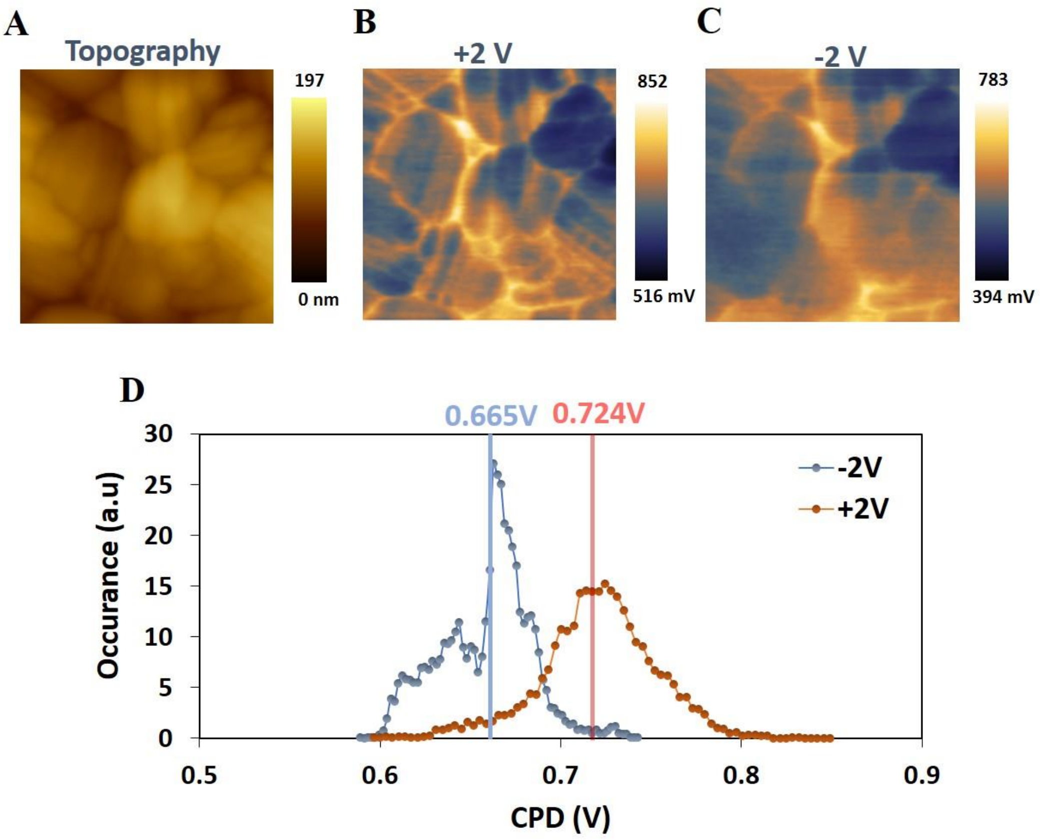


**Supplementary Figure 10. KPFM measurement under positive and negative bias.** (A) Topography and CPD spatial maps over an area of 1 μm with (B) +2 V and (C) -2 V applied to the tip under 500 nm with 0.3 W/cm$^2$ illuminations of the samples consisting of FTO/bl-$TiO_2$/mp-$TiO_2$/perovskite and the FTO layer is grounded. (D) CPD distributions and vertical lines are medians for each graph. Here, we used a novel two-pass Kelvin mode in which the first pass scans the topography and the second pass measures the CPD. Bias voltages are applied during the first pass scans.

**Supplementary Note 1**

Light and bias can cause both reversible and irreversible chemistry and structural rearrangements, depending on the magnitude of applied bias or light intensity.[1] There is partial irreversible/reversible chemistry or structural rearrangement exists which could be responsible for phase segregation or externally stimulated ion migration through the material by bias or light.

Firstly, looking at the broaden peak near 38 degrees, Hoke et al showed that such peak broadening under the light originates from phase segregation and it is mostly reversible after dark condition for 5 mins.[2] For our sample, it was almost reversible after the illumination, and partially irreversible for applied bias. This could be due to a permeant damage caused by a strong ion migration. Next, in the new peak near 45 degrees, it can be seen that the peak does not change under the light, however, the new peak extends from 43 degrees (300) and has a maximum at around 44 degrees. Similar results are observed from reversible phase segregation reports[2] which stated that the strain is driven by phase segregation under light illumination, which can cause peak splitting and broadening.[2,3] Consequently, it is evident that changed peaks are not caused by permanent damage of the material.

In addition, XRD patterns are analyzed to see if there is any damage due to bias and light. If there was an irreversible damage or amorphisation, there should be an increase for the $PbI_2$ peak near around 13 degrees[4], because halide perovskites usually decompose into MAI or FAI, and $PbI_2$.[5] The $PbI_2$ peak does not show noticeable change, however, the intensities of the presented perovskite peaks significantly change, which indicates that the crystallinity of the film has been partly modified due to the perturbations. This could potentially be originating from mostly reversible phase segregation (after dark condition for 5 mins[2]) or reversible conversion mentioned in earlier reports[6]. Therefore, we think that it is not possible to conclude a complete transition to the amorphous phase or irreversible damage to the sample.

Note that we see damage to the material only at a certain high bias range especially for above – 4 V.[1] All our measurements are however only performed at lower bias where such damage can be avoided. Although the PL spectral result (Fig. S1) did not show clear indication of phase segregation, we now think that the phase segregation can occur due to the bias which alters the XRD patterns.

**Supplementary Note 2**

In Williamson-Hall analysis, pure strain broadening is expected to result in a straight line through the origin without intercept.[7] In principle, a smooth line indicates isotropic broadening of each peak regardless of the plane orientation.[7] However, this is not observed in our results which mean that our film undergoes anisotropic peak broadening. In this case, it is more appropriate to use the broadening functions of separate effects with families of higher order reflections.[8-10] Previously, it has been reported that halide perovskites can have anisotropic strain depending on the plane orientation.[11,12] Accordingly, we have analysed the strain data considering families of strongly oriented (110) and (100) lattice planes. (2θ: 14°, 20°, 28°, 40.5°, and 43°) Based on above arguments and references, we exclude peaks at 24, 38 and 44 degrees because they are not the same family as (100) or (110), and also because they might have contributions from phase segregation as discussed above.

## Supplementary References


1 J. S. Yun, J. Seidel, J. Kim, A. M. Soufiani, S. Huang, J. Lau, N. J. Jeon, S. I. Seok, M. A. Green, A. Ho-Baillie. Critical Role of Grain Boundaries for Ion Migration in Formamidinium and Methylammonium Lead Halide Perovskite Solar Cells *Adv. Energy Mater.* **6**, 1600330 (2016).

2 E. T. Hoke, D. J. Slotcavage, E. R. Dohner, A. R. Bowring, H. I. Karunadasa, M. D. McGehee. Reversible Photo-Induced Trap Formation in Mixedhalide Hybrid Perovskites for Photovoltaics. *Chem. Sci.* **6**, 613-617 (2015).

3 D. J. Slotcavage, H. I. Karunadasa, M. D. McGehee. Light-Induced Phase Segregation in Halide-Perovskite Absorbers. *ACS Energy Lett.*, **1**, 1199 (2016).

4 Y. Wu, A. Islam, X. Yang, C. Qin, J. Liu, K. Zhang, W. Penga, L. Han. Retarding the Crystallization of $PbI_2$ for Highly Reproducible Planar-Structured Perovskite Solar Cells via Sequential Deposition. *Energy Environ. Sci.* **7**, 2934 (2014).

5 J. S. Yun, J. Kim, T. Young, R. J. Patterson, D. Kim, J. Seidel, S. Lim, M. A. Green, S. Huang, A. Ho-Baillie. Humidity-Induced Degradation via Grain Boundaries of $HC(NH_2)_2PbI_3$ Planar Perovskite Solar Cells. *Adv. Funct. Mater.* **28**, 1705363 (2018).

6 Y. Yuan, Q. Wang, Y. Shao, H. Lu, T. Li, A. Gruverman, J. Huang. Electric-Field-Driven Reversible Conversion Between Methylammonium Lead Triiodide Perovskites and Lead Iodide at Elevated Temperatures. *Adv. Energy Mater.* **6**, 1501803 (2016).

7 G. K. Williamson, W. H. Hall. X-ray Line Broadening From Filed Aluminium And Wolfram. *Acta Metall.* **1**, 22 (1953).

8 T. Ungár, A. Borbély. The Effect of Dislocation Contrast on X-ray Line Broadening: A New Approach to Line Profile Analysis. *Appl. Phys. Lett.* **69**, 3173 (1996).

9 T. Ungár, G. Tichy. The Effect of Dislocation Contrast on X-Ray Line Profiles in Untextured Polycrystals. *Phys. Sol. A* **171**, 425 (1999).

10 B. E. Warren. X-Ray Measurement of Stacking Fault Widths in fcc Metals. *J. Appl. Phys.* **32**, 2428 (1961).

11 L. Leppert, S. E. Reyes-Lillo, J. B. Neaton. Electric Field- and Strain-Induced Rashba Effect in Hybrid Halide Perovskites. *J. Phys. Chem. Lett.* **7**, 3683 (2016).

12 X. Zheng, C. Wu, S. K. Jha, Z. Li, K. Zhu, S. Priya. Improved Phase Stability of Formamidinium Lead Triiodide Perovskite by Strain Relaxation. *ACS Energy Lett.* **1**, 1014 (2016).
